# A 3D Statistical Shape Model of the Right Ventricular Outflow Tract in Pulmonary Valve Replacement Patients Post-Surgical Repair

**DOI:** 10.3390/jcdd11100330

**Published:** 2024-10-17

**Authors:** Liam Swanson, Raphaël Sivera, Claudio Capelli, Abdulaziz Alosaimi, Dariusz Mroczek, Christopher Z. Lam, Andrew Cook, Rajiv R. Chaturvedi, Silvia Schievano

**Affiliations:** 1 Institute of Cardiovascular Science, University College London, London WC1E 6BT, UK; 2 Great Ormond Street Hospital for Children, London WC1N 3JH, UK; 3 Labatt Family Heart Centre, Division of Cardiology, The Hospital for Sick Children, Toronto, ON M5G 1X8, Canada; 4 Department of Paediatrics, University of Toronto, Toronto, ON M5G 1X8, Canada; 5 Department of Diagnostic Imaging, The Hospital for Sick Children, Toronto, ON M5G 1X8, Canada; 6 Department of Medical Imaging, University of Toronto, Toronto, ON M5T 1W7, Canada

**Keywords:** right ventricular outflow tract, percutaneous pulmonary valve implantation, statistical shape modelling, congenital heart disease

## Abstract

Assessment of the right ventricular outflow tract and pulmonary arteries (RVOT) for percutaneous pulmonary valve implantation (PPVI) uses discrete measurements (diameters and lengths) from medical images. This multi-centre study identified the 3D RVOT shape features prevalent in patients late after surgical repair of congenital heart disease (CHD). A 3D RVOT statistical shape model (SSM) was computed from 81 retrospectively selected CHD patients (14.7 ± 6.8 years) who required pulmonary valve replacement late after surgical repair. A principal component analysis identified prevalent shape features (modes) within the population which were compared with standard geometric measurements (diameter, length and surface area) and between sub-groups of diagnosis, RVOT type and dysfunction. Shape mode 1 and 2 represented RVOT size and curvature and tapering and length, respectively. Shape modes 3–5 related to branch pulmonary artery calibre, conical vs. bulbous RVOTs and RVOT curvature, respectively. Tetralogy of Fallot, transannular patch type and regurgitant RVOTs were larger and straighter while conduit and stenotic types were longer and more cylindrical than other subgroups. This SSM analysed the main 3D shape features present in a population of RVOTs, exploiting the wide 3D anatomical information provided by routine imaging. This morphological information may have implications for PPVI patient selection and device design.

## 1. Introduction

Congenital heart disease (CHD), defined by structural birth defects of the cardiac chambers, valves or great arteries, is the most common congenital abnormality, affecting millions of babies worldwide each year [1,2]. A recent meta-analysis, studying CHD prevalence between 1970 and 2017, identified tetralogy of Fallot (ToF) and pulmonary stenosis as the 4th and 5th most common CHD sub types, accounting for 10% of CHD diagnosis [2]. These diseases predominantly affect the pulmonary valve, the right ventricular outflow tract and the pulmonary trunk (henceforth together referred to as the RVOT) and require corrective open-heart surgery in the first year of life. Repeated surgeries and interventions over the course of the patients’ lives are necessary to address residual or recurrent hemodynamic burdens, or to replace materials and devices implanted during initial surgical repair due to their limited lifespan [3]. Within current treatment strategies for RVOT dysfunction, percutaneous pulmonary valve implantation (PPVI) plays an important role thanks to its minimally invasive nature that does not compromise safety or effectiveness [4]. The advent of innovative stent solutions and valve technologies has expanded the pool of patients who can benefit from this minimally invasive approach [5]. Nevertheless, patient selection remains challenging due to the extreme heterogeneity and complexity in the RVOT morphology observed in these patients late after initial surgical repairs. This complexity calls for the development of new techniques and instruments for clinicians to assess anatomy to select the optimal device for patients. Improved morphological characterisation would also guide the design of new valved stent designs.

The RVOT anatomical variations present in patients with RVOT dysfunction following surgical CHD repair were investigated by our group in 2007 [6]. Detailed measurements of RVOT diameters and lengths from 3D cardiovascular magnetic resonance (CMR) reconstructions showed heterogenous characteristics and identified five morphological classes. The most prevalent class (pyramidal morphology, 49%) related to the presence of a transannular patch (TAP), deemed unsuitable for PPVI with the Melody device (Medtronic, Minneapolis, MN, USA). The study highlighted the importance of a thorough RVOT anatomical assessment for PPVI device design and patient selection. Nevertheless, most of the RVOT shape information was lost in this study as only a subset of dimensions at a limited number of locations along the RVOT were measured, mostly manually.

The analysis of the shape of cardiovascular structures, including atria [7], atrial appendages [8,9], aorta [10,11] and left and right ventricles [12,13,14], has steadily progressed from the use of isolated measurements of lengths, diameters, angles and ratios towards greater adoption of 3D statistical shape modelling (SSM) methods. These allow the analysis of 3D shape features prevalent in a population of objects by computing a template shape—representative of the mean of the population—and the shape variations present in the population—defined as deformations of this template [11]. Thus, SSMs can be used both to visualise and to quantify the anatomical characteristics of a cohort of CHD shapes, to explore how shape features are associated with population demographics [15] and clinical outcomes [16] using traditional statistics, and to support the design of new treatments and solutions [17].

The aim of this study is to present an SSM of a population of 3D RVOT anatomies from CHD patients who require pulmonary valve replacement, to provide insight into the key 3D shape features present in the population. The proposed SSM may support development of future PPVI device designs and patient selection.

## 2. Materials and Methods

### 2.1. Patient Population and CMR Image Processing

CMR angiography images of patients referred for pulmonary valve assessment at Great Ormond Street Hospital for Children (GOSH) and Toronto SickKids Hospital between 2003 and 2018 were reviewed from previously collated databases. Patients from these two centres were selected for this study if they had previously had surgical repair and at the time of the scan qualified for pulmonary valve replacement, due to moderate or severe pulmonary regurgitation, stenosis or mixed valve disease. Cause for exclusion included anatomical abnormalities such as interrupted branch pulmonary arteries (PAs), low image quality, presence of artefacts from implanted devices and lack of surgical history details. Demographics and surgical history were retrieved for the selected patients, who were categorised by sex, primary diagnosis (ToF, ToF + pulmonary atresia, other), RVOT repair type (TAP, conduit, RVOT patch, other) and RVOT dysfunction (purely regurgitant, mixed, purely stenotic).

**Figure 1:**
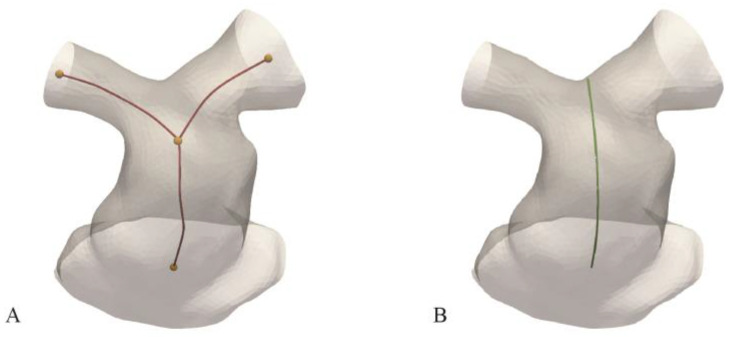
Example of patient RVOT processed surface including (**A**) bifurcating centreline and corresponding landmark locations, and (**B**) single line from inlet to bifurcation saddle for perimeter derived diameter calculation.

The medical image software Mimics (v24.0 Materialise, Leuven, Belgium) was used by a single user for CMR image segmentation to extract the region of interest through semi-automatic thresholding and manual cleaning tools. The region of interest was defined proximally by the axial plane located at the most superior point of the tricuspid valve annulus, and distally at the points of secondary bifurcations of the pulmonary arteries. Each segmentation was exported as a surface mesh to 3-matics (Materialise) for smoothing and re-meshing to improve mesh uniformity and quality. Using VMTK (Vascular Modelling Toolkit, Orobix, Bergamo, Italy) and Meshmixer (AutoDesk Inc., San Francisco, CA, USA), the centrelines were calculated, and the proximal and distal regions of interest were clipped with planes orthogonal to the centreline, at the start of the contractile RV myocardium and, for the PA branches, at the points with distance equal to the proximal RVOT maximum inscribed sphere radius from the bifurcation point (Figure 1).

The following measurements and features were extracted from each geometry:

- 4 landmarks (orange dots in Figure 1A) corresponding to the ends of the centrelines, and the bifurcation point.
- Surface area (SA)
- RVOT centreline length (L_RVOT_) between the proximal and bifurcation landmark (Figure 1A)
- Perimeter-derived RVOT diameters at 1 mm increments along the line which originated at the proximal opening and terminated at the bifurcation saddle (Figure 1B). The perimeters from those cross-sections that did not intersect an opening were considered for analysis. An average diameter (D_AVE_) was calculated for each patient.

All surfaces were rigidly registered to the subject with the geometric parameters closest to the mean of the population as the initial reference [18] using a least-squares approach, first on the 4 landmarks to avoid misregistration of the LPA and RPA openings when distances between surfaces were large, and then on the centrelines to account for disparities between subjects’ curvatures and calibres. Correspondence in the final registration was ascertained through an iterative closest point method to avoid the need for point-to-point correspondence. To balance computational demand and model fidelity, the registered shapes were all re-meshed to a target edge length of 2 mm, calculated according to the sensitivity method established by Bruse et al. [18] which used the subject with the smallest SA to define the upper limit of re-meshing based on <0.5% change to its SA.

### 2.2. Statistical Shape Modelling

The Deformetrica package (www.deformetrica.org, accessed on 29 March 2023) [19,20] was used to construct the SSM non-parametrically from the computational surface meshes of the population without need for anatomical landmarks. The template shape, representing the mean of the population, was computed, and each input geometry was represented as a deformation applied to the template toward each subject, thus allowing standard statistical analysis on the population of shape deformation vectors. The framework by Bruse et al. was followed to validate each step, including kernel width optimisation, population registration validation, template validation and k-fold cross-validation [18].

The subject with SA closest to the mean of the population was used as an initial template (T_0_). The initial, coarse kernel widths for the Deformetrica framework were estimated and used to compute a new template (T_1_) more representative of the population mean [18]. Due to the large variation between the subjects, a stepped, multiscale approach was implemented to gradually deform the template towards the target shape and avoid introduction of reconstruction anomalies: in the first step, large kernel width and low control point density were used to marginally deform the template towards the population. These results initialised a new computation with stepped-down kernel widths and higher control point density, repeated twice to achieve a final converged template, T_2_. The same multiscale approach was applied through the rest of model development. The accuracy of the reconstructions was assessed on the median of average distance between the surface points of the reconstructed population vs. those of the input population, compared to the mean voxel size of the CMR images.

The new template T_2_ centrelines and corresponding landmarks were computed and used for iterative registrations of the population until the compactness (as defined by Styner et al. [21]) of the model decreased. The resulting template T_3_ was validated by comparing SA, L_RVOT_ and D_AVE_ between the template shape and the average of these parameters in the population. A 9-fold cross-validation procedure was implemented to assess if some subjects carried undue weight in the template computation. A unique set of 9 randomly chosen shapes were removed from the population 9 times and the template recomputed for each resulting sub-group, ensuring that each subject was left out exactly once throughout the procedure. The resulting 9 template shapes were compared by measuring the average distance between the corresponding surface points on each new template and T_3_.

Principal component analysis (PCA), a dimensionality reducing technique used to project high-dimensional data into a reduced number of main components (shape modes in SSM) was conducted to assess the shape variability in the population. The shape modes capturing ≥5% shape variance were visualised at ±2 standard deviations (SD) from the mean. Subjects’ shape vectors—representing the projection of the shapes on each PCA shape mode—were analysed in comparison with geometric parameters (SA, L_RVOT_ and D_AVE_) to describe the shape modes, and with demographics and clinical information to interrogate any underlying correlations between shape and function.

### 2.3. Statistical Analyses

All statistical tests were performed in Python using the SciPy v1.10.1 library and using a significance level of 5%. The Anderson–Darling test was used to test the normality of continuous demographic (age) and geometric (SA, L_RVOT_ and D_AVE_) variables of the cohort. Non-normally distributed variables were described by their median and interquartile ranges (IQR) and normally distributed variables by their mean ± standard deviation (σ).

One-way ANOVA tests were conducted to assess if any differences existed between the distributions of the continuous variables (including the shape vectors of the PCA shape modes) of the sub-groups in each demographic category (sex, diagnosis, RVOT repair type and RVOT dysfunction). If a difference was observed, post hoc independent Student’s *t*-tests with unequal variance or Wilcoxon Rank Sum (for normally or non-normally distributed variables, respectively) were used to indicate significant differences between each pair of sub-groups.

Bivariate Pearson correlation analyses were used to test for correlations between continuous variables. Correlations were tested between each geometric variable and the logarithm of age, and the shape vectors of each of the PCA shape modes and the logarithm of age, to assess for any influence of age with shape or size. Correlations between the shape vectors of the PCA shape modes and geometric measurements were assessed to support the findings of the SSM and glean a quantitative description of the shape modes.

## 3. Results

### 3.1. Patient Population and CMR Image Processing

A total of 100 patients were initially selected from databases incorporating the two centres. Amongst these, 19 cases were excluded because of branch PA stents (n = 5), disconnected/interrupted left PA (n = 2), poor image quality (n = 6), a lack of surgical data (n = 5) and recent pulmonary valve replacement (n = 1). The characteristics of the remaining patients (n = 81, 49% female, age at scan = 14.5 (IQR = 6.13) years) are shown in Table 1, with further information about diagnosis and dysfunctions in Table 2. ToF (including ToF + pulmonary atresia) diagnoses were the most prevalent in the cohort (n = 72) and most of these patients had a TAP repair (n = 49). Patients with TAP repairs (n = 50) presented with pure regurgitation (n = 43) or mixed type RVOT dysfunction (n = 7) at the time of CMR assessment. Conduit repairs (n = 19) occurred equally between ToF, ToF + pulmonary atresia and other diagnoses, and mostly presented with mixed disease (n = 9), followed by regurgitant (n = 7) and purely stenotic (3) RVOTs. All patients who had purely stenotic RVOT disease had had a conduit repair. The small number of purely stenotic RVOT dysfunction cases excluded them from statistical testing; the data for these stenotic cases are shown on the plots.

**Figure 2:**
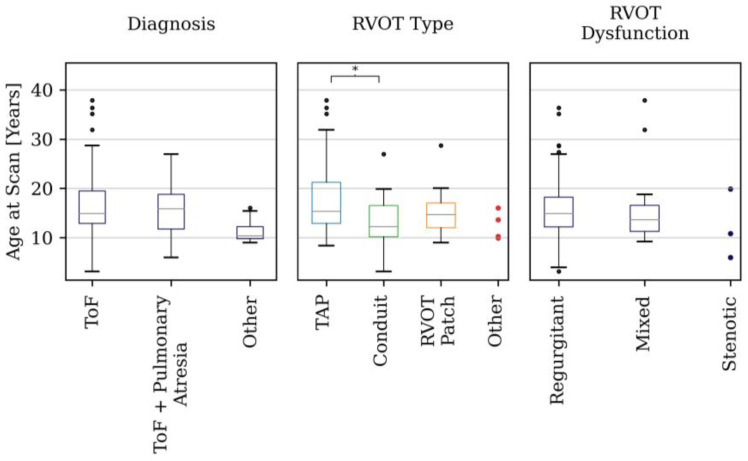
Box plots of the age distribution in the different sub-group categories. * denotes statistically significant differences.

Figure 2 shows the box plot distributions of age within each demographic category. Patients with conduit repairs were younger than patients with TAP repairs (*p* = 0.01). No statistically significant differences in age were otherwise found between sub-groups of sex, diagnosis or RVOT dysfunction.

**Figure 3:**
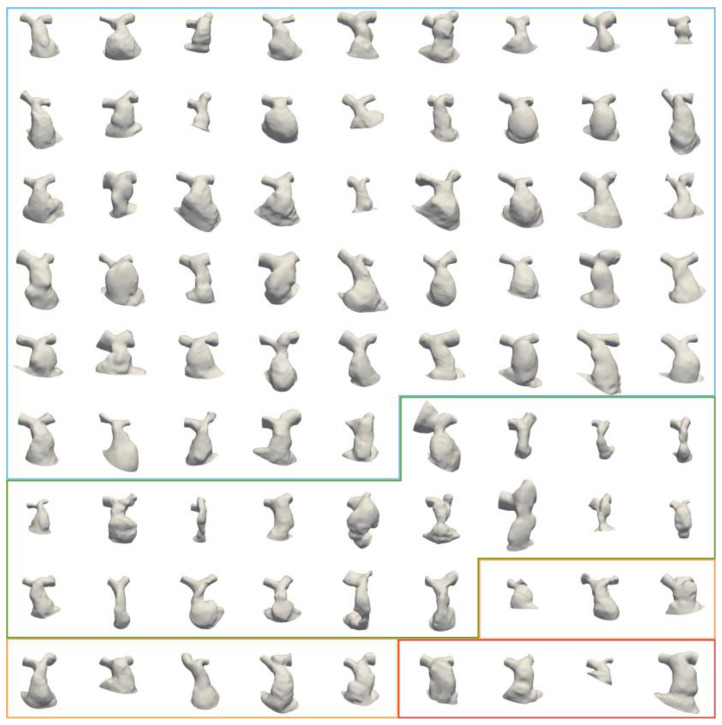
Population of input RVOT surfaces arranged by RVOT type (blue = TAP, green = conduits, orange = RVOT Patch, red = others), ordered by age within each group from left to right and top to bottom.

The average voxel dimension of the CMR image dataset was 1.2 mm × 1.2 mm × 1.6 mm. Figure 3 shows the population of RVOT shapes after CMR image reconstruction and surface processing. The average geometric measurements in the populations were as follows: SA = 7785 ± 2269 mm^2^, L_RVOT_ = 59 ± 12 mm and D_AVE_ = 29 ± 6 mm.

**Figure 4:**
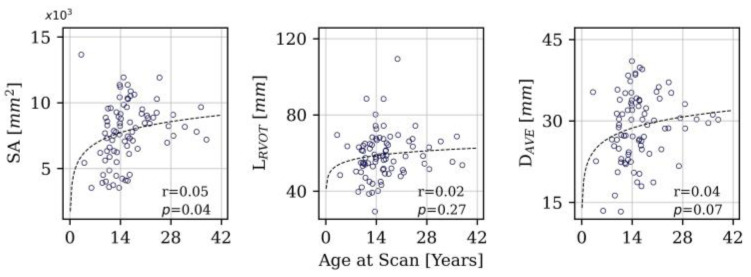
Surface area (SA), RVOT centreline length (L_RVOT_) and RVOT average diameter (D_AVE_) plotted against age, including Pearson r and statistical significance.

**Figure 5:**
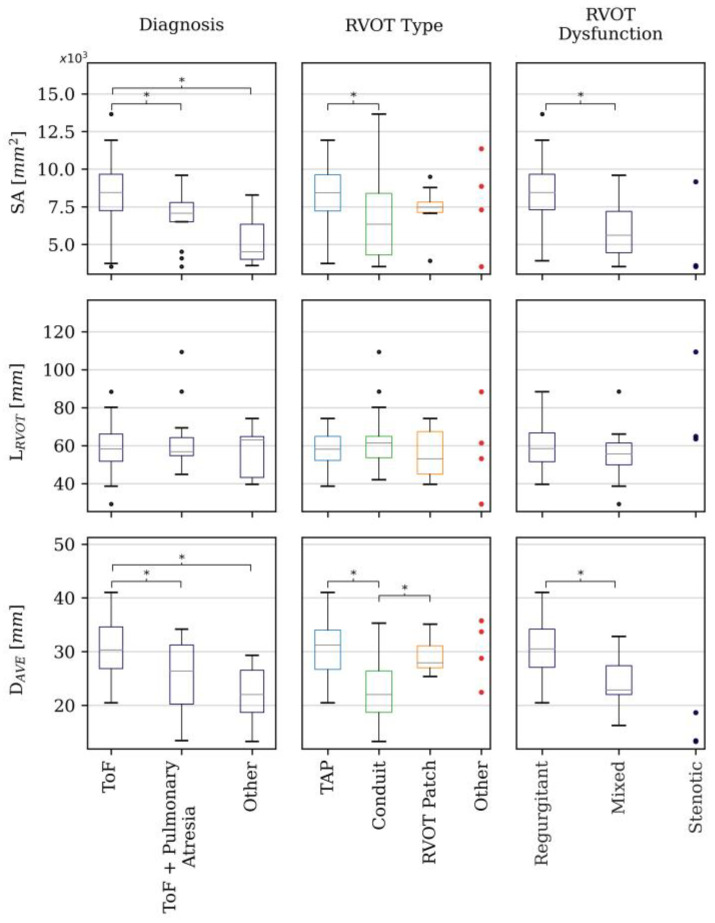
Box plots of SA, L_RVOT_ and D_AVE_ distributions in the different subgroups per each studied category (primary diagnosis, RVOT type and RVOT dysfunction). * denotes statistically significant differences.

Figure 4 shows that there was negligeable correlation between all geometric variables and the logarithm of age. There was no significant age difference between male and female cases (*p* = 0.3) and so this was not included in the figure. Figure 5 compares the distribution of the geometric variables in the other sub-groups: In the diagnosis category, ToF patients had larger RVOTs than ToF + pulmonary atresia patients (SA *p* = 0.03 and D_AVE_ *p* = 0.01) and ‘other’ type diagnoses (SA *p* < 0.001 and D_AVE_ *p* < 0.001). TAP RVOTs were larger than conduits based on SA (*p* = 0.03) and D_AVE_ (*p* < 0.001) measurements, whilst RVOT-patch RVOT types had larger average diameters (*p* = 0.008) than conduit RVOT types. Purely regurgitant RVOTs were found to have larger SA (*p* < 0.001) and average diameter (*p* < 0.001) than mixed dysfunction RVOTs. L_RVOT_ showed no significant difference between sub-groups in any category. There were no statistically significant differences between male and female sub-groups for each geometric variable (SA: *p* = 0.89, D_AVE_: *p* = 0.65, L_RVOT_: *p* = 0.48)

### 3.2. Statistical Shape Modelling

**Figure 6:**
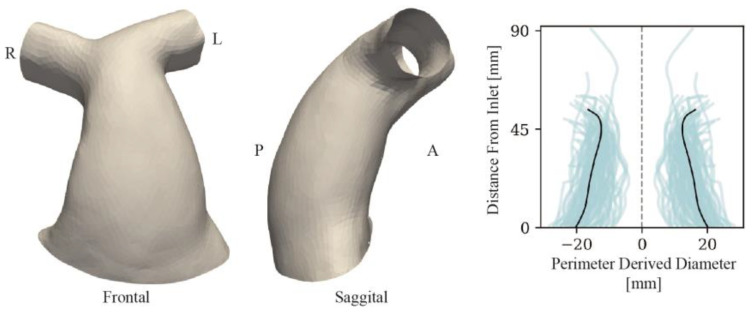
Computed template of the population and, on the right, RVOT perimeter-derived diameter plots for each case (blue) and the template (black) from the inlet to the branch pulmonary artery bifurcation.

The computed SSM template (Figure 6) qualitatively resembled an RVOT with little evidence of stenosis or dilation. It was neither overly flared, cylindrical or bulging, finding a middle-ground between native and conduit geometries. The comparison of SA, L_RVOT_ and D_AVE_ variables between the mean of the population and the template showed small absolute differences (0.1%, 5.9% and 2.0%, respectively). The reconstructions of the subject surfaces from the template resulted in a reconstruction error with a median distance between surfaces of 0.29 mm (IQR = 0.10 mm), smaller than the average CMR voxel dimension.

**Figure 7:**
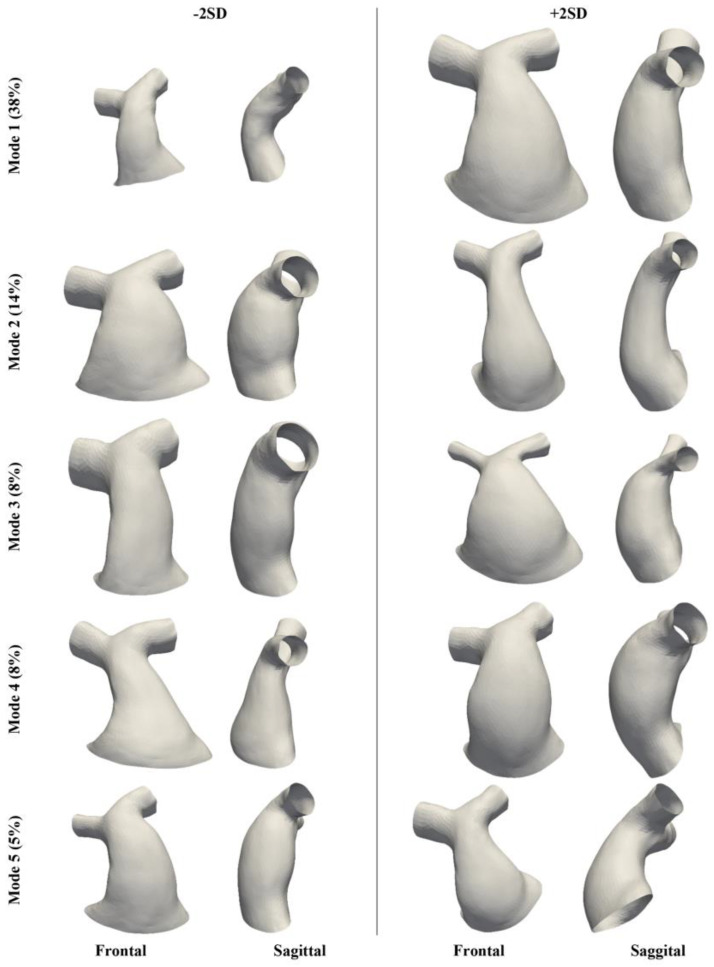
Frontal and sagittal view of the first five shape modes from −2 to +2 SD. The % of shape variance represented in each mode is reported in brackets.

**Figure 8:**
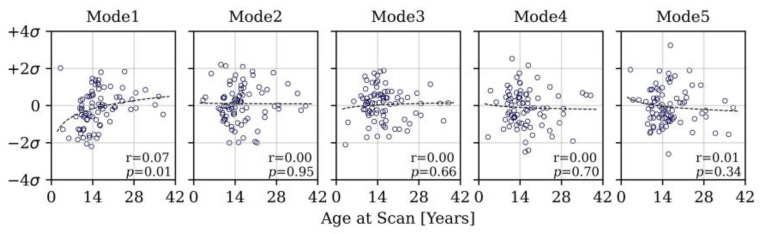
Relationship between the shape vectors in each mode and age, showing no correlation.

**Figure 9:**
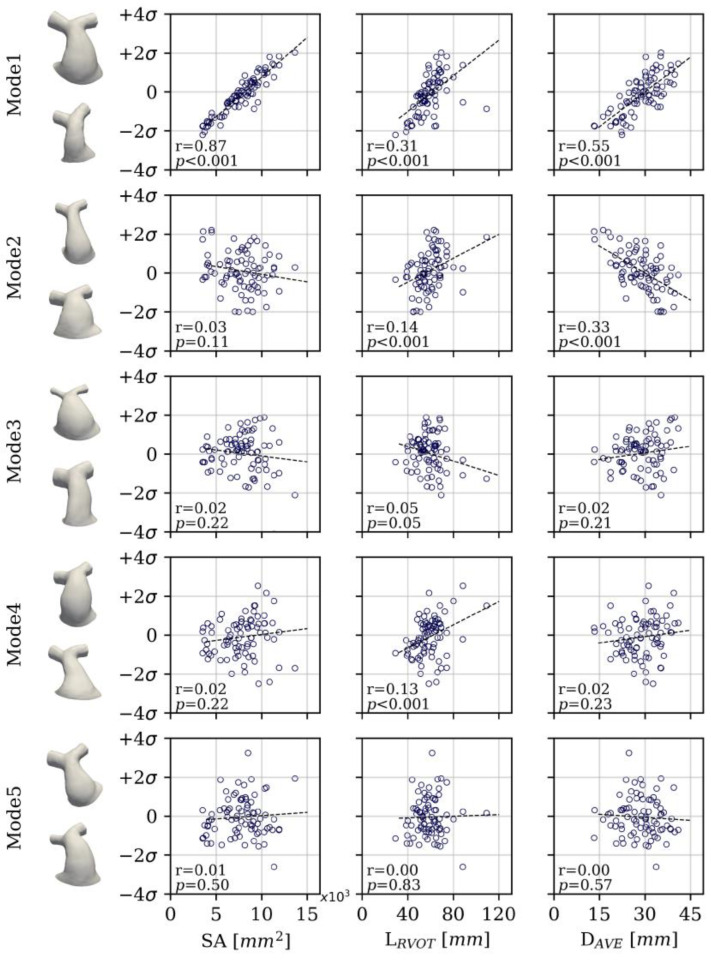
Morphological measurements of SA, RVOT centreline length (L_RVOT_) and RVOT average diameter (D_AVE_) compared to shape vectors in modes 1–5.

**Figure 10:**
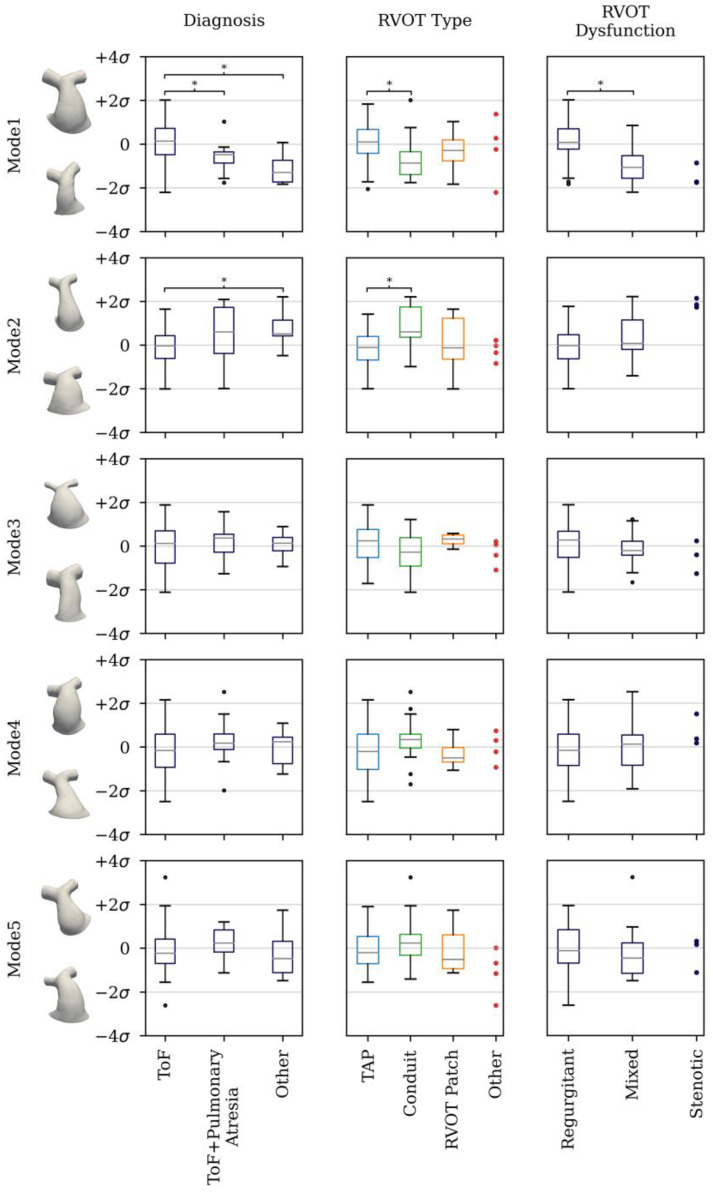
Box plots of the distributions of each shape mode in the subgroups of the studied categories. * denotes statistically significant differences.

The PCA reduced the dimensionality of the space and provided a more compact description of shape such that 85% of the overall population shape variation could be described with just 10 shape modes. The first five modes accounted for 73% of cumulative variance. From the sixth mode onwards, modes individually accounted for <5% of overall shape variance and changes to shape features became more localised. Figure 7 visualises the shape variations captured by the first five modes at +/−2 SD, whilst Figure 8, Figure 9 and Figure 10 present the correlations of shape vectors in each mode with age, geometric variables and a comparison of the distributions between sub-groups in each category, respectively. There was no correlation between shape vectors of the population and the logarithm of age in any shape mode (Figure 8), and no distinction was found between males and females.

The first mode accounted for 38% of the variance in the population, and predominately captured overall size (Figure 7). This is supported by the positive correlations of the shape vectors with measures of SA (r = 0.87, *p* < 0.001), L_RVOT_ (r = 0.31, *p* < 0.001) and D_AVE_ (r = 0.55, *p* < 0.001) (Figure 9). Additional to overall size, there is evidence of changes in the RVOT curvature both in the frontal plane, from a more hemodynamically efficient connection between the trunk and the left PA (−2 SD) compared to the trunk and the right PA (+2 SD), and in the sagittal plane, from a bent (−2 SD) to a straighter outflow tract (+2 SD). The shape vectors of the patients with a ToF diagnosis in the first mode were significantly higher than those of ToF + pulmonary atresia (*p* = 0.006) and ‘other’ (*p* < 0.001) diagnoses patients. Furthermore, TAP RVOT types had larger mode 1 shape vectors than conduit RVOT types (*p* < 0.001), and regurgitant RVOT dysfunction had larger mode 1 shape vectors than mixed (*p* < 0.001) RVOT dysfunction types (Figure 10).

Mode 2 represented 14% of the shape variance, capturing shape changes from a short, stocky and bulbous shape (−2 SD) to a longer, slenderer and tapering RVOT (+2 SD). A moderately strong, negative correlation existed between the mode 2 shape vector and the RVOT average diameter (r = 0.33, *p* < 0.001) but with no other geometric variable, reflecting mode 2 shape changes independent of overall size (Figure 9). The mode 2 shape vectors of ‘other’ diagnoses were higher than ToF diagnoses (*p* = 0.02), and conduit RVOT type shape vectors were higher than both TAP (*p* < 0.001) and ‘other’ (*p* = 0.02) RVOT types (Figure 10).

There were no meaningful correlations between the shape vectors of modes 3–5 and geometric variables, reflecting shape changes independent of overall size. Mode 3 accounted for 8% of the total variance and qualitatively represented shape variations between a regular, cylindrical RVOT shape (−2 SD) and a highly flared RVOT (+2 SD) with a stenotic bifurcation area, and in the calibre of the branch PAs, from widely patent (−2 SD) to narrow PAs (+2 SD), with striking contrasting proportions between the RVOT and the PA calibre. Mode 4 captured 8% of the total variance, with an interesting transition from a conical RVOT with curvature in the frontal plane favouring the right PA and a relatively straight shape in the sagittal plane (−2 SD) to a more bulbous RVOT with a restricted proximal and distal section, a straighter configuration in the frontal plane and a pronounced curvature in the sagittal plane (+2 SD). Mode 5 had a small contribution to the variance (5%) of shape in the population. Figure 7 shows a change in shape and orientation of the inlet to the RVOT, from right and elliptical (−2 SD) to left and circular (+2 SD), impacting the RVOT curvature in the frontal plane. There were no significant differences in the shape vector distributions of modes 3–5 amongst the diagnosis, RVOT type and dysfunction subgroups.

## 4. Discussion

In this study, an SSM was computed from 3D CMR images of a cohort of 81 CHD patients from two centres, requiring pulmonary valve replacement. The SSM identifies the 3D shape features and size of the RVOT which were prevalent and representative in this population. In contrast to previously proposed classification methods, the key shape features were identified by considering the entirety of the 3D shape information. Indeed, compared to previous studies, new features were highlighted that may be important for design considerations for future PPVI devices and patient selection.

The cohort included mostly ToF diagnoses and TAP RVOT types, and had a wide range of anatomical heterogeneity. The SSM, validated by comparison with traditionally measured geometric parameters, was able to capture 73% of the variability of the represented complex morphologies with five principal shape components. It showed, first and foremost, and as expected, the overall size variations in the population and that TAP repairs (mostly associated with ToF) resulted in larger RVOTs than conduits. However, it also highlighted differences in shape. TAP RVOTs tended to have a greater degree of tapering than conduit repair types. Conduits presented a strong bend in the sagittal plane and curvature that favours connection to the left PA, as opposed to the straighter TAP RVOTs, which had a favourable connection to the right PA and prominent angulation towards the left PA. Conduits were also longer and more slender compared to all TAP RVOTs.

Principal shape components greater than the fifth component had less than 5% contribution to shape variance, indicating more localised shape effects. These are less relevant for device design and patient selection considerations. The first five shape modes ensured that 73% of the overall shape variance was addressed. The distribution of variance between these reflects the fact that accounting for the range of sizes is indeed the biggest contribution to the range of shapes, but only to a limited extent: the first mode accounts for 38% of the variance. Other features may be relevant for device design, as shown by the first five components.

The spectrum of RVOT morphology present in patients late after surgical repair of CHD was already described by our group using a set of linear measurements [6]. However, these discrete parameters only partially capture the heterogeneity and otherwise large amount of 3D anatomical information routinely provided by 3D imaging. In addition, they rely on manual selection of anatomical landmarks and relevant cross-sections. This is the case in routine clinical practice for CMR and computed tomography (CT) imaging assessment of the RVOT for PPVI selection [22], but the analysis is prone to errors particularly in the highly variable set of shapes such as those studied. This is particularly because the valve cusps are often not visible, the boundaries between the right ventricular cavity and outflow tract, as well as between the outflow tract and the valve structures, cannot be identified, and the relative angles between the branch PAs and the main trunk make it difficult to determine the precise position of the bifurcation point.

The proposed SSM allows a population study that considers the entirety of the RVOT 3D shapes to objectively identify and isolate the main shape features in the population and, importantly, quantitatively study them in comparison to other geometric or demographic characteristics. It is strengthened by its unsupervised computation of point-to-point surface correspondence, which removes the need for manual anatomical landmarking or manual selection of specific cross-sections, prone to operator errors. Thus, the SSM provides a more objective characterisation of key shape features than previous studies.

Similar to other study findings [3,23,24,25], TAP RVOT type patients in our cohort presented for pulmonary valve replacement later than homograft cases. However, age—as well as sex—did not correlate with RVOT size or any of the identified shape features. For example, the youngest subject of the conduit subgroup (green boxing of Figure 3) also had the largest SA, driven by the aneurysmal dilation of the RVOT and right PA, whilst older patients presented with considerably smaller overall RVOTs. To emphasise this, shape mode 1, which captures global size information and indeed presents significant correlation with all geometric parameters, did not display a correlation with age. Thus, the shape and size heterogeneity found in the population and the SSM are considered independent of the age of the patients, and are instead a result of the initial disease, surgical repair approach, subsequent remodelling and resultant dysfunction.

The length of the RVOT, despite its wide range in the population, did not differ significantly between the subgroups in each category, whilst the average diameter along the RVOT did. ToF and purely regurgitant patients had larger average diameters compared to ToF + pulmonary atresia cases and mixed valve disease respectively. Conduit RVOT types had smaller diameters compared to all TAP and RVOT-patch RVOT types. However, important overlapping in diameters exists between repaired RVOT types, with a few conduits that presented large average diameters and, vice versa, some TAP RVOTs with rather small diameters. Indeed this overlap makes sense, as off-label PPVI devices initially approved for conduit RVOT types were found to fit some smaller TAP RVOT type implantation sites successfully [26,27].

Together with several other factors, anatomical measurements guide the selection process for PPVI, to guarantee safe device anchoring, avoid oversizing that could lead to wall rupture, and achieve good valve function with no residual stenosis, leakage or creation of paravalvular leaks.

Current PPVI devices present straight, cylindrical or ‘hourglass’ shapes, with a varying range of diameters and lengths, but fixed ratios between inlet and outlet, relative to the valve diameters. This study has highlighted more complex shape features that include curvatures, tapering and different length/diameter relationships at inlet/outlet, currently not accounted for. For example, shape modes 1 and 2, capturing the majority of the shape variations in the cohort, indicate that larger RVOTs tend to have increased tapering. Patients with large RVOTs may not find adequate fit among the available designs because devices which have a large enough inlet diameter to guarantee anchoring and adequate seal against paravalvular leakage may cause wall erosion at the outlet due to the oversized distal ring.

Although more comprehensive measurements have been introduced with the advent of new solutions for native RVOTs, current device sizing and selection remain predominantly based on a set of RVOT diameter and length measurements at discrete locations along the implantation site [28,29]. Selection criteria based on varying combinations of these measurements are stipulated by device manufacturers, but each prioritises different, fewer or more measurements.

The variety of approaches is seen when one examines PPVI devices available or in development today. The maximum inner diameter of the landing zone is the key dimension for Melody (Medtronic, 18–22 mm diameters [30]) and Sapien (Edwards, 16–28 mm based on transoesophageal echocardiography or 18.6–29.5 mm based on area-derived diameters calculated from CT [31]) devices. The size selection for the Harmony device (Medtronic), indicated for dilated native RVOTs, relies on maximal perimeter-derived RVOT diameters (TPV22: proximal 23–39 mm, valve > 22 mm, distal 22–28 mm; TPV25: proximal 32–48 mm, valve > 25 mm, distal 25–38 mm) and lengths (TPV22 ≥ 55 mm; TPV25 ≥ 51 mm) taken from ECG-gated CTA at end diastole [32]. The Pulsta valve (TaeWoong Medical Co, 18–28 mm diameter) is reported to be chosen to be 2–4 mm larger than the narrowest area of the implantation site, and equal to or slightly larger than the mean RVOT size [33]. The Venus *p* valve device size is selected based on the MRI and bi-plane angiography for balloon interrogation. The valve diameter is chosen by oversizing 2–4 mm from the size of the balloon waist up to maximum RVOT diameter of 32–33 mm and the length is chosen by the distance from the valve annulus to the pulmonary artery bifurcation [34]. The Med-Zenith PT-Valve relies on systolic and diastolic perimeter-derived diameter measurements at several levels in the RVOT to assess interference between device and implantation site. In early studies, the device was oversized by 4–8 mm at the anchoring site; however, exact sizing guidelines are yet to be published [35]. The Edwards Alterra Prestent selection is dependent on the RVOT landing zone diameters (27–38 mm) typically measured during systole [36].

Indeed, the limitations of using discrete diameter and length measures of the RVOT was demonstrated during the screening process for the early feasibility study of the Harmony device. In this study, with a single-size device (TPV22), only 21 (32%) patients met anatomical criteria for implantation out of the 66 patients who underwent CT scans (out of 270 patients who were pre-screened by CMR), and, in one of these, the device migrated proximally during the procedure [37]. Despite the varied and numerous multi-modal measurements, there remains enough uncertainty about the suitably of the patients’ RVOT shapes for PPVI that clinicians continue to seek more shape descriptors [38,39] like those by Schievano et al. [6].

As demonstrated by this SSM, diameters and lengths alone do not capture the global shape information. It is evident from the shape features identified in this SSM that single measurements of diameter or gross simplifications such as average diameter, previously described, are insufficient to assess the device selection. Selection criteria of new devices such as the Pulsta and Venus *p*-Valve, described above, are driven by local diameter measurements. This approach in large and tapered RVOTs puts selection at risk of a false positive for device selection, as discussed previously. Particularly in devices which have such a wide range of diameter and length combinations, it is appropriate that their device selection criteria are expanded to capture key dimensions in other areas of the RVOT. In general, the SSM and shape features identified here motivate the extension of device selection criteria towards 3D superposition or, more ideally, complete simulated deployment. In this way, all of the 3D information is retained and clinicians can assess the length, diameter and tapering suitability between various devices and the RVOT of interest.

The implantation site anatomical complexities and heterogeneities highlighted by the SSM translate to PPVI design challenges, as a small family of device designs/sizes must be able to adequately fit most of these shape features to benefit the largest number of patients. The first two shape modes captured 52% of the shape variations in the populations. Although modes 3–5 individually accounted for only 8, 8 and 5% of the overall shape variations, respectively, they still jointly held 21% of the overall shape variation, notwithstanding the 27% captured by the remaining shape modes.

### Limitations

The main limitation of this study is the retrospective nature of the selected cohort with no proportional representation of the groups within each category of repair, diagnosis, regurgitation severity or valvular disease type. However, the studied population provides a fair snapshot of patients who require a new pulmonary valve, with the majority being ToF diagnoses, with TAP augmentation repairs, resulting in regurgitant valve disease, representative of the period during which this cohort underwent surgery (late 1900s and early 2000s). As such there is a co-dependency between sub-groups of different categories, and the causal effect on shape based on the groups within each category is not explicit and not tested within the bounds of this study design. Hence, the analysis is an observational study of the shapes that present in patients requiring a new pulmonary valve with no specific claims of any single group.

For the cohort selected in this study, only information relative to the time of CMR assessment was available, with no follow-up data. However, a similar methodology to that presented in this paper could be leveraged in the future for a retrospective analysis of patients who underwent PPVI, with the aim of extracting shape features mostly associated with the use and suitability of each of the PPVI devices available nowadays on the market. This would allow one to extract shape biomarkers that could help improve patient selection and clinical outcomes.

The uncertainties related to the segmentation process were minimised by a single user analysing the images and by quantifying surface distances before and after each step. Furthermore, the decision not to remove size yielded insight into general size differences between conduits and TAP repairs—important in PPVI device design and selection—but in studying shape alone might dominate other shape features which underlie the population and quantitatively reduce their perceived importance. Further development of the study could seek to control for size to dig deeper into shape alone and understand groupings of shapes, respectively.

Finally, partial least square regression modelling or different classifications techniques, such as unsupervised hierarchical clustering, could be implemented to extract shape patterns most related to relevant clinical parameters/outcomes, or to reveal groupings of anatomical features within the patient population that could drive personalisation of treatment strategies for precision medicine.

## 5. Conclusions

In this study, an SSM tool was implemented to study the morphological features present in a population of surgically repaired RVOTs indicated for a new pulmonary valve. This unsupervised method, which requires no anatomical landmarking, allowed analysis of the wide range of sizes and shape characteristics of the population, fully exploiting the detailed 3D anatomical information provided by routine CRM and summarising the main morphological features in a small number of 3D shape modes. This comprehensive 3D shape assessment of the RVOT, in combination with routinely acquired functional data, could improve patient selection for PPVI and guide the design of new devices.

## Figures and Tables

**Table 1:** Patient characteristics. ToF = Tetralogy of Fallot, TAP = transannular patch, RVOT = right ventricular outflow tract. * Other diagnoses: truncus arteriosus (4), absent pulmonary valve (1), double outlet right ventricle (1), isolated pulmonary stenosis (1), aortic atresia (1) and transposition of the great arteries (1). ** Other repairs: valve-preserving repair (2), main pulmonary artery patch (1) and RVOT + main pulmonary artery patch (1).

Group	Subgroup	n = 81
Sex	Female	40 (49%)
	Male	41 (51%)
Diagnosis	ToF	59 (73%)
	ToF + Pulmonary Atresia	13 (16%)
	Other *	9 (11%)
RVOT Type	TAP	50 (62%)
	Conduit	19 (23%)
	RVOT Patch	8 (10%)
	Other **	4 (5%)
RVOT Dysfunction	Regurgitant	61 (75%)
	Mixed	17 (21%)
	Stenotic	3 (4%)

**Table 2:** Cohort breakdown according to RVOT type by diagnosis and RVOT dysfunction.

Group	Subgroup	TAP (n = 50)	Conduit (n = 19)	RVOT Patch (n = 8)	Other Repair (n = 4)
Diagnosis	ToF	46	6	3	4
	ToF + Pulmonary Atresia	3	7	3	0
	Other	1	6	2	0
RVOT Dysfunction	Regurgitant	43	7	8	3
	Mixed	7	9	0	1
	Stenotic	0	3	0	0

## Data Availability

The raw data supporting the conclusions of this article will be made available by the authors on request due to privacy and ethical reasons.
